# Comparative transcriptomics of the pheromone glands provides new insights into the differentiation of sex pheromone between two host populations of *Chilo suppressalis*

**DOI:** 10.1038/s41598-020-60529-x

**Published:** 2020-02-26

**Authors:** Shuang Guo, Zhong Tian, Wei-Li Quan, Dan Sun, Wen Liu, Xiao-Ping Wang

**Affiliations:** Hubei Key Laboratory of Insect Resources Utilization and Sustainable Pest Management, College of Plant Science & Technology, Huazhong Agricultural University, Wuhan, 430070 PR China

**Keywords:** Evolution, Molecular biology

## Abstract

Reproductive isolation between different host populations is often based on intraspecific sex pheromone differences. The mechanisms underlying these differences have not been thoroughly elucidated to date. Previous studies suggested that *Chilo suppressalis* has differentiated into rice and water-oat host populations, and these two populations manifest clear differences in sex pheromone titer and mating rhythm. Hence, this moth is an ideal model to investigate the endogenous mechanisms of intraspecific reproductive isolation. Here, we identified a series of putative genes associated with sex pheromone biosynthesis based on the *C. suppressalis* pheromone gland transcriptome data. Transcripts of most genes were at higher level in the rice population. Then we obtained 11 pivotal differentially expressed genes (DEGs). The expression levels of these DEGs exhibited a distinct increase in the rice population. Moreover, we also observed the expression rhythm of these DEGs is discrepant between two host populations. Our study offers a new understanding to elucidate the mechanisms of intraspecific reproductive isolation.

## Introduction

Reproductive isolation is generally regarded as an important step to promote sympatric speciation^1–3^. In many moths, reproductive isolation is common to maintain reproductive isolation between host populations through intraspecific differences in sex pheromone communication^4–8^. Previous studies revealed that in addition to the differences in the ratios of sex pheromone compounds, variations in sex pheromone titer can also lead to strong reproductive isolation among different populations^9–11^. In insects, the production of sex pheromone often exhibits a diel rhythm, which is closely related to the mating activity rhythm^12,13^. When moths differ in their sex pheromone production rhythms, they are selected to be sexually active at different times, which can cause extensive communication interference, eventually resulting in intraspecific reproductive isolation^5,14–16^. Hence, understanding the endogenous mechanisms of the variations in sex pheromone would help to elucidate the occurrence of intraspecific reproductive isolation.

In several moths, the regulation of sex pheromone biosynthesis has been shown to involve a series of biochemical enzymes^17–19^. Hence, the divergence in these enzymes may lead to variations of pheromone production. With the development of molecular biology techniques, a large number of putative genes involved in sex pheromone biosynthesis have been identified, including acetyl-CoA carboxylase, fatty acid synthase, fatty acid transport proteins, acyl-CoA oxidases, desaturases, and aldehyde reductase^20–24^. Several studies showed that these genes generally exhibited diel expression rhythms in moths^25,26^. Thus, the difference in expression levels and rhythm of genes related to sex pheromone biosynthesis probably cause the variations in sex pheromone production among different populations.

*Chilo suppressalis* Walker (Lepidoptera: Crambidae) is an economically important pest in Asia, which appeared in rice and water-oat populations^27–30^. As early as the 1970s-1980s, Japanese scholars identified three active components in the sex pheromone gland extract of females, including Z9-16Ald, Z11-16Ald, and Z13-18Ald^10^. They also demonstrated the sex pheromone titer was of a higher level in the rice population than in the water-oat population^10^. Meanwhile, the two host populations showed significant differences in diel mating rhythm^16^, which may be dominated by variation in sex pheromones titer rhythms^22,31^. Thus, *C. suppressalis* is an ideal model to investigate intraspecific differences in sex pheromone communication. We proposed that the differential expression of putative sex pheromone biosynthetic genes might lead to divergence in sex pheromone production.

In this study, we obtained the putative genes related to sex pheromone biosynthesis and analyzed expression patterns of the key differentially expressed genes (DEGs) in the two host populations, basing on the pheromone gland transcriptome data. There were divergences in expression patterns between the two populations of *C. suppressalis*, which is consistent with sex pheromone titer and mating rhythm between these two populations in the previous researches. Our study provides a new insight into the endogenous mechanism of intraspecific reproductive isolation.

## Results

### Sex pheromone gland transcriptome assembly, functional annotation and DEG analysis in *C. suppressalis*

To elucidate the molecular basis of the variation in sex pheromone, we performed RNA-Seq to quantify gene expression between the rice and water-oat populations. After removing low-quality reads, 23 to 33 million clean reads from each sample were obtained for further analysis (Table 1). The final non-redundant dataset contained 169477 unigenes with an N50 of 1830 bp and a mean length of 1115 bp (Table S1). The sequences of all the reads have been deposited in the NCBI SRA database with the accession number, SRP162187. Unigenes in the transcriptome were annotated according to seven databases. A total of 61,422 (36.24%), 28,805 (16.99%), 21,036 (12.41%), 42,162 (24.87%), 47,893 (28.25%), 48,322 (28.51%), and 30,528 (18.01%) unigenes could be mapped to the NR, NT, KO, Swiss-Prot, PFAM, KOG and GO databases, respectively (Table S2). The percent of annotated genes assigned to the biological processes, cellular components and molecular function categories was 48.5%, 30.4% and 21.0%, respectively, based on the GO database^32^ (Fig. S1).

**Table 1:** Summary of *C. suppressalis* pheromone gland sequencing reads and quality metrics.

Sample	Raw Reads	Clean Reads	Clean Bases	Q20	GC Content
R0^a^_1	33,299,218	32,148,426	4.82 G	97.16%	43.04%
R0_2	29,545,158	28,379,906	4.26 G	97.60%	42.88%
R0_3	30,256,192	29,104,104	4.37 G	97.58%	42.71%
R3_1	33,268,874	31,460,310	4.72 G	96.88%	42.98%
R3_2	26,126,644	25,183,518	3.78 G	96.85%	43.15%
R3_3	27,058,962	26,096,298	3.91 G	97.14%	42.74%
R6_1	29,501,788	28,422,308	4.26 G	97.08%	42.50%
R6_2	32,295,978	31,014,090	4.65 G	97.03%	43.83%
R6_3	24,932,768	23,954,566	3.59 G	97.02%	42.32%
W0_1	29,814,248	28,002,098	4.20 G	96.57%	44.59%
W0_2	27,343,088	25,831,456	3.87 G	96.66%	42.56%
W0_3	27,862,866	26,854,950	4.03 G	97.04%	42.88%
W3_1	29,736,018	28,114,434	4.22 G	96.79%	43.22%
W3_2	27,946,514	26,971,444	4.05 G	97.19%	43.07%
W3_3	28,831,070	27,203,918	4.08 G	96.82%	42.60%
W6_1	30,226,446	28,957,384	4.34 G	97.19%	44.26%
W6_2	27,521,754	26,507,980	3.98 G	97.14%	42.85%
W6_3	28,076,838	27,099,430	4.06 G	97.28%	42.97%

^a^R and W stands for rice population and water-oat population; 0, 3 and 6 stand for 0 h, 3 h and 6 h after the onset of scotophase.

**Figure 1:**
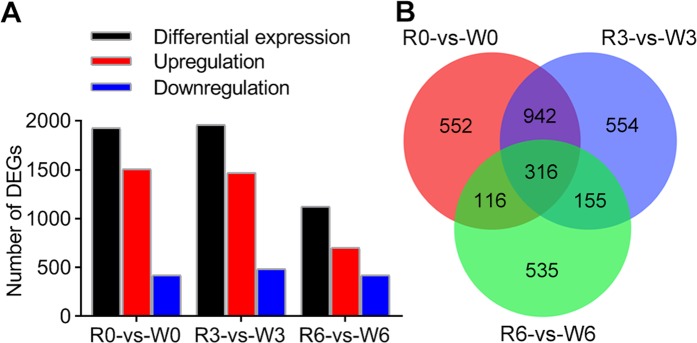
Analysis of DEGs in the pheromone gland transcriptome between rice (R) and water-oat populations (W) of *C. suppressalis*. (**A**) Distribution of upregulated and downregulated DEGs in each comparison; (**B**) Venn diagram of DEGs in different comparisons among the groups. Upregulations indicate that the genes have a higher expression in the rice population compared to the water-oat population.

Among the DEGs between the two host populations of *C. suppressalis*, 1926 genes were differentially regulated between R0 and W0, including the upregulation of 1505 genes and the downregulation of 421 genes. Similarly, a total of 1957 DEGs, including 1471 upregulated and 486 downregulated, were detected when comparing R3 and W3 (Fig. 1A, Table S3). A significant decrease in the DEGs between R6 and W6 was observed, with 702 genes upregulated and 420 genes downregulated (Fig. 1A, Table S3). These results showed that most DEGs are upregulated in the rice population. Among these DEGs, 316 unigenes were commonly differentially expressed in response to the three comparison groups (Fig. 1B). Meanwhile, through KEGG pathway enrichment analysis, we found that the amino sugar and nucleotide sugar metabolism pathways and steroid hormone biosynthesis were significantly enriched (Table 2). This suggests that energy regulation may be an important factor in the process of sex pheromone production.

**Table 2:** KEGG pathway enrichment of DEGs in the pheromone gland transcriptome between the rice and water-oat populations of *C. suppressalis*.

Enriched KEGG pathway	Gene number	*P*-value^b^
**R0-vs-W0**^**a**^		
Ribosome	40	5.85E-03
Purine metabolism	33	9.83E-05
Pyrimidine metabolism	28	6.39E-07
Cell cycle	23	4.53E-05
Glutathione metabolism	10	4.81E-02
Protein export	9	9.85E-04
Arginine and proline metabolism	9	4.10E-02
**R3-vs-W3**		
Cell cycle	6	1.61E-03
Purine metabolism	6	2.51E-02
Pyrimidine metabolism	5	1.05E-02
Cysteine and methionine metabolism	4	5.03E-03
Nicotinate and nicotinamide metabolism	3	7.14E-03
TGF-beta signaling pathway	3	1.96E-02
Protein export	2	4.55E-02
**R6-vs-W6**		
Retinol metabolism	7	3.88E-04
Cell cycle	6	4.67E-02
Steroid hormone biosynthesis	5	2.29E-03
Amino sugar and nucleotide sugar metabolism	5	2.76E-02
Arginine and proline metabolism	4	4.68E-02
Linoleic acid metabolism	3	3.66E-03
Protein export	3	3.54E-02
Nicotinate and nicotinamide metabolism	3	4.86E-02
D-Glutamine and D-glutamate metabolism	2	6.64E-03
Mucin type O-Glycan biosynthesis	2	3.26E-02

^a^R and W stands for rice population and water-oat population; 0, 3 and 6 stand for 0 h, 3 h and 6 h after the onset of scotophase. ^b^*P*-value <0.05 is considered significant.

### The expression patterns of putative genes associated with sex pheromone biosynthesis between the rice and water-oat populations in *C. suppressalis*

**Figure 2:**
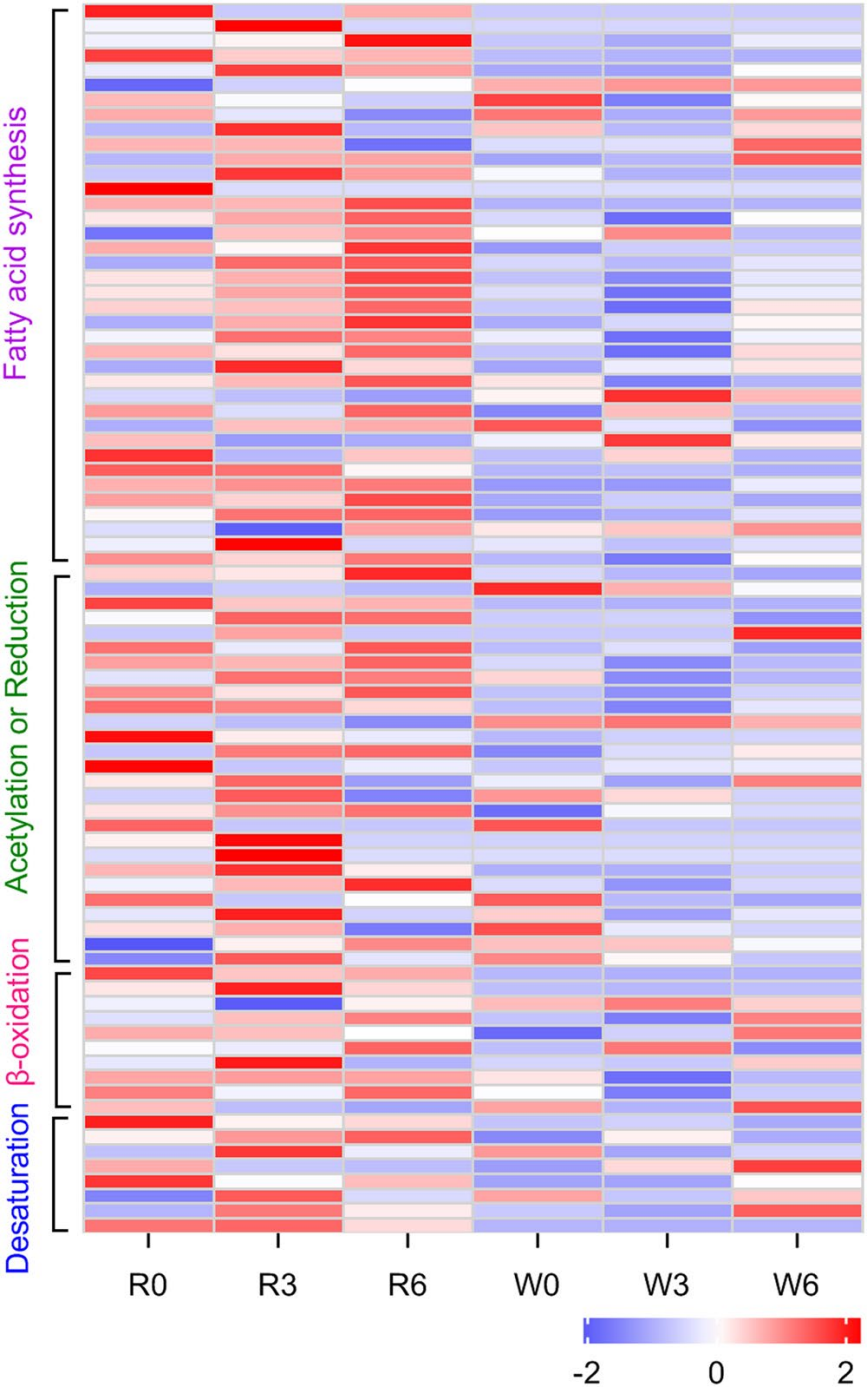
Heatmap analysis of expression patterns of putative genes associated with sex pheromone biosynthesis at 0 h, 3 h and 6 h after the onset of scotophase between rice (R) and water-oat populations (W) in *C. suppressalis*. Prior to analysis, FPKM values of the genes were Z-score standardized. The heatmap was plotted using GraphPad Prism 8 (GraphPad Software Inc., San Diego, CA, https://www.graphpad.com). Blue and red colors in the heatmap correspond to low and high relative gene expression level, respectively.

**Figure 3:**
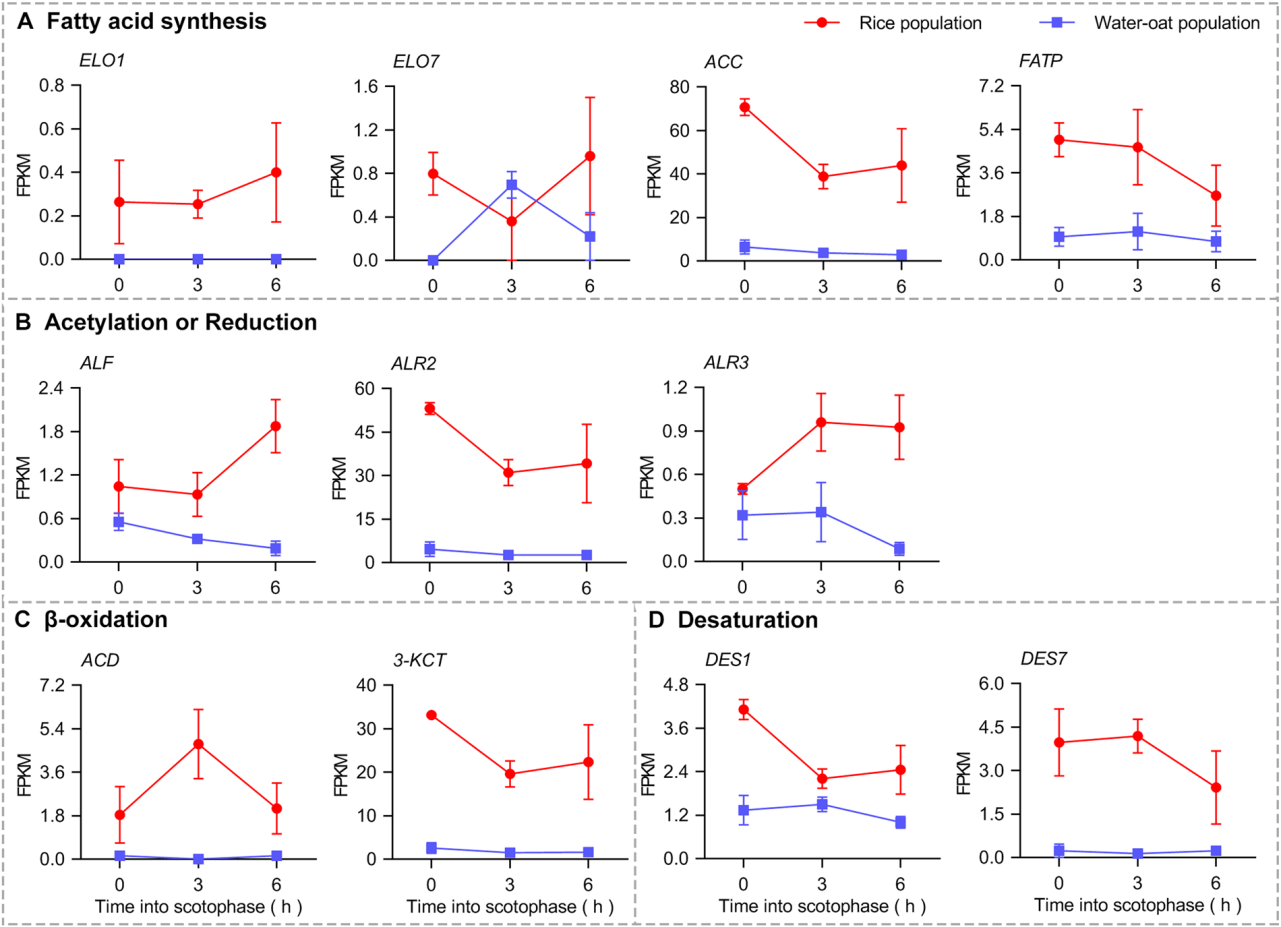
Expression levels of putative DEGs associated with sex pheromone biosynthesis between the rice populations (R) and water-oat populations (W) in *C. suppressalis*, as determined by FPKM values. FPKM values are represented as the means ± standard error of mean (SEM) based on three biological replicates.

The enzymatic reactions that occur during sex pheromone biosynthesis include various processes^21,33^. Based on functional annotation and homology analysis, we identified a series of putative genes associated with sex pheromone biosynthesis and investigated the overall expression kinetics of these genes. These genes were involved four biochemical processes, including fatty acid synthesis, desaturation, β-oxidation and reduction reactions. Transcripts of these genes were have higher levels in the rice populations (Fig. 2), which is in agreement with the sex pheromone titer data^10^. Next, we obtained 11 key putative DEGs associated with sex pheromone biosynthesis, including 2 *ELO*, 1 *ACC*, 1 *FATP*, 1 *ALF*, 2 *ALR*, 1 *ACD*, 1 *3-KCT* and 2 *DES* genes (Table 3). The results showed that the expression levels of 11 genes exhibited a significant elevation in the rice population (Fig. 3). Moreover, *ELO7*, *ACC*, *FATP*, *ALR2*, *3-KCT*, *DES1* and *DES7*, had higher expression at 0 h or 3 h after the onset of scotophase in the rice population (Fig. 3A–D). Additionally, *ELO7*, *FATP*, *ACD* and *DES1* had higher expression at 3 h or 6 h after the onset of scotophase in the water-oat population (Fig. 3A,B,D). This suggests the different expression peaks of these genes may lead to the differentiation in mating rhythm between the two populations in *C. suppressalis*.

**Table 3:** The putative DEGs associated with sex pheromone biosynthesis in *C. suppressalis*.

Gene (Abbreviation)	Query ID	R0-vs-W0^a^		R3-vs-W3		R6-vs-W6	
		| log2 ratio |	*P*-value^b^	| log2 ratio |	*P*-value	| log2 ratio |	*P*-value
*Elongation of very long chain fatty acids 1* (*ELO1*)	Cluster-28055.77797	Infinity	1.00	Infinity	2.70 E-02	Infinity	0.37
*Elongation of very long chain fatty acids 7* (*ELO7*)	Cluster-28055.94024	Infinity	1.52E-03	0.96	1.00	2.25	1.00
*Acetyl-CoA Carboxylase* (*ACC*)	Cluster-28055.115852	3.64	1.00E-08	3.37	1.36E-04	4.02	0.13
*Fatty acid transport protein* (*FATP*)	Cluster-28055.89390	2.50	2.12E-04	2.06	0.99	1.81	1.00
*Acetyltransferase1* (*ALF*)	Cluster-28055.50331	1.01	1.00	1.59	1.00	3.26	2.11E-02
*Aldo-Ketose Reductase2* (*ALR2*)	Cluster-28055.89156	3.68	1.16E-12	3.61	7.87E-07	3.78	0.21
*Aldo-Ketose Reductase3* (*ALR3*)	Cluster-28055.76800	0.77	1.00	1.52	0.7741	3.43	2.09E-03
*Acyl-CoA dehydrogenase* (*ACD*)	Cluster-28055.81963	3.79	1.00	Infinity	1.76E-08	3.99	0.66
*3-ketoacyl-CoA thiolase* (*3-KCT*)	Cluster-28055.79228	3.85	2.12E-14	3.73	1.91E-07	3.87	0.15
*Desaturase 1* (*DES1*)	Cluster-28055.80420	1.74	2.35E-02	0.60	1.00	1.35	0.86
*Desaturase 7* (*DES7*)	Cluster-28055.25897	4.29	2.86E-03	4.94	1.42 E-06	3.32	1.00

^a^R and W stands for rice population and water-oat population; 0, 3 and 6 stand for 0 h, 3 h and 6 h after the onset of scotophase. ^b^*P*-value <0.05 is considered significant.

**Figure 4:**
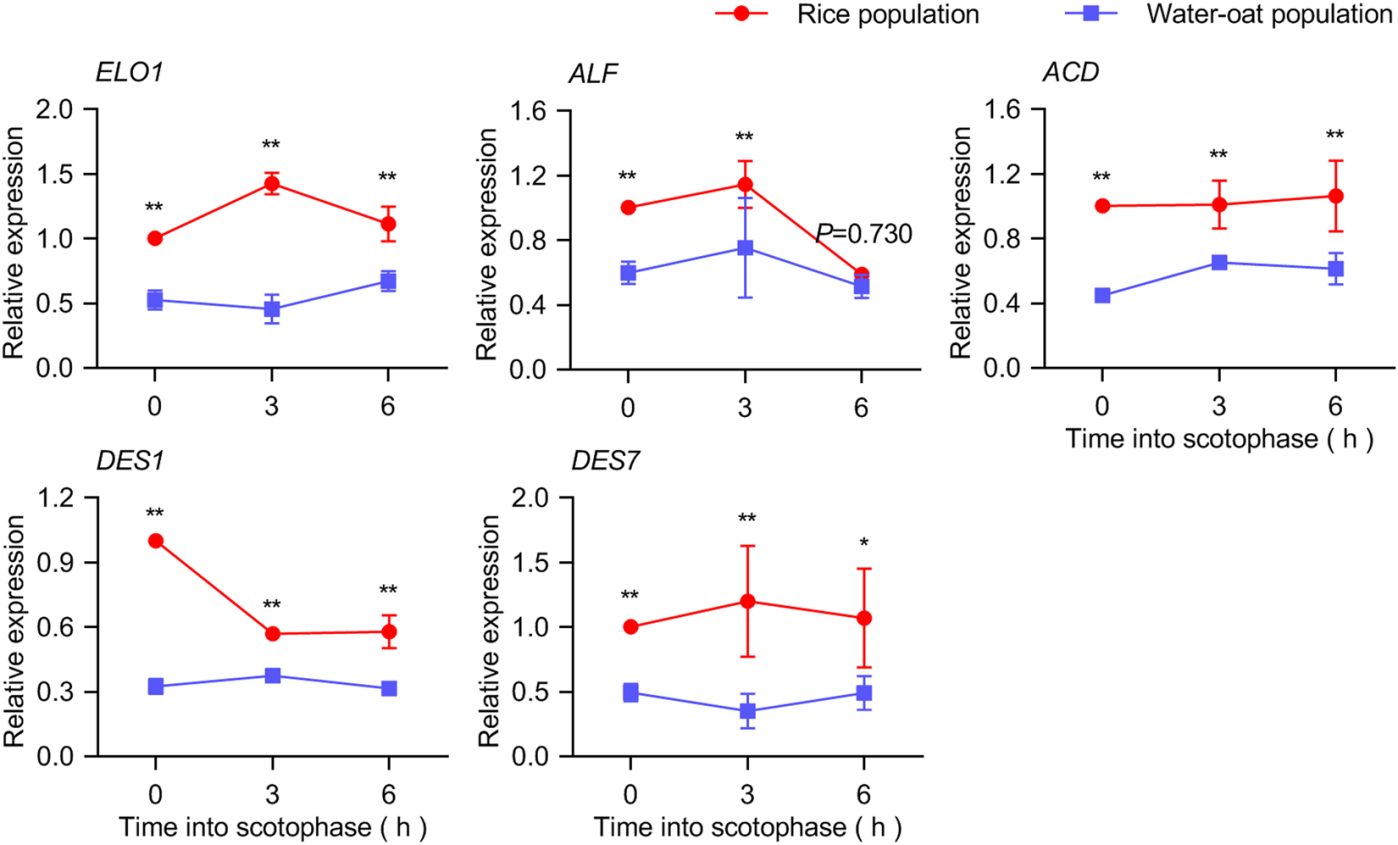
Relative expression of putative sex pheromone biosynthetic genes between the rice populations (R) and water-oat populations (W) in *C. suppressalis*, as determined by RT-qPCR. Relative expression values are represented as the means ± standard error of mean (SEM) based on three biological replicates. Significance analysis of the relative gene expression between the two populations was carried out by independent sample t-tests, *P <0.05, **P <0.01.

To verify the reliability of this transcriptome database, we randomly select 5 DEGs, *ELO1*, *ALF*, *ACD*, *DES1* and *DES7*, to be validated through RT-qPCR (Fig. 4). This validation result is coincided with the RNA sequencing regarding the gene expression levels at the 3 stages.

## Discussion

In moths, variations in sex pheromone are an important component in the evolution of the sex pheromone communication system^10^, which is an initial step towards pre-mating reproductive isolation in host populations^5^. In this study, we revealed the differences in the expression patterns of putative sex pheromone biosynthetic genes in rice and water-oat populations of *C. suppressalis*, which is of great significance for our understanding of the endogenous mechanisms of intraspecific reproductive isolation

Here we obtained 11 key putative DEGs involved in sex pheromone biosynthesis with higher expression in the rice population (Table 3). Among them, ELO, ACC and FATP are all highly important enzymes in the fatty acid synthesis, which are essential precursors in the process of sex pheromones production^34–37^. For example, ACC as an important regulator mediated *de novo* biosynthesis of fatty acid from acetyl-CoA^38^, and it has been proved to be a key component involved in sex pheromone biosynthesis in *Helicoverpa armigera*^39^. The extracellular fatty acids can also be derived, transported, absorbed and activated by FATP^40^. Additionally, acetyl-CoA can be used as substrate and fatty acids can continue to extend the carbon chain under the action of ELO^36,41^. The increased expression of these genes in the rice population suggested that the long-chain fatty acids were derived not only from intracellular sources but were also supplied energy from extracellular substrates at the same time (Fig. 3A), so that there was more increased substrate content for sex pheromone biosynthesis in the rice population. Desaturases are key enzymes that introduce double bonds into pheromone molecules^42,43^. It has been confirmed in studies of *B. mori*^44^, *Plutella xylostella*^45^, *Ostrinia* moths^46,47^ and *Thaumetopoea pityocampa*^48^ that desaturases play an important role in sex pheromone biosynthesis. In the sibling species of leafroller moths, *Ctenopseustis* and *Planotortrix*, differential expression of the desaturase genes resulted in interspecific sex pheromone differences^49^. In our research, we found that the expressions of *DES1* and *DES7* in rice population were higher than that in water-oat population (Fig. 3D). This result showed that desaturation would be more effective in increase of sex pheromone biosynthesis in the rice population. ACD catalyses the β-oxidation (shortening of carbon chains) of long-chain fatty acids and ALR can catalyse fatty acids into fatty aldehydes^33,50^. In our study, we speculate that the high expression of *ALR2*, *ALR3* and *ACD* could expedite the β-oxidation or reduction process and elevate the sex pheromone biosynthesis in the rice population. The sex pheromone titer measurement would be required to further validate.

On the other hand, we found that there were differences in the expression rhythms over time of the putative genes related to sex pheromone synthesis between the two host populations (Fig. 3). Among them, *ACC*, *FATP*, *ELO7* and *DES1* were synchronously highly expressed in the early stages of scotophase in rice population and in the late stages of scotophase in water-oat population (Fig. 3A,D). This difference potentially gives rise to variations in sex pheromone production, which adapts to the courtship and mating rhythm of different populations^50,51^.

## Conclusions

Taken together, these results revealed that there are divergences in expression levels and rhythms of putative genes associated with sex pheromone biosynthesis in the two host populations. Further explorations would be needed to assess the specific functions of these genes in sex pheromone production. Our study provides a genetic basis for understanding mechanism of intraspecific reproductive isolation.

## Materials and Methods

### Insects

Approximately 1000 and 700 overwintering larvae of *C. suppressalis* were collected from rice (113° 57′E, 30° 29′N) and water-oat fields (114° 16′E, 30° 28′N), respectively, in Wuhan City, China in December 2015. The collected overwintering larvae of *C. suppressalis* were reared at 28 ± 1 °C, with LD 15: 9 h (light: dark photoperiod) and RH = 80 ± 5% in an insectarium^52^. The mating rhythm of emerged moths in the overwintering generation was observed to identify the rice and water-oat populations^16,53,54^. The offspring of the two host populations were fed on an artificial diet^55^, and the 2-day-old virgin female moths were used in the subsequent experiments.

### Sex pheromone gland sample preparation for RNA extraction

Our previous study have shown that the mating activity reached different peaks at 3 h and 6 h after the onset of scotophase in the rice and water-oat populations, respectively^16^. We thus prepared pheromone glands samples for RNA-Seq at 0 h (R0, W0), 3 h (R3, W3) and 6 h (R6, W6) after the onset of scotophase in two host populations. The pheromone glands were extruded by gently exerting force on the abdomen of 2-day-old virgin female moths randomly for samples. Pheromone glands were extracted and immediately frozen in liquid nitrogen and stored at −80 °C for RNA extraction. Samples of *C. suppressalis* pheromone glands were collected from groups of 30 females, and three biological replicates were used for each analysis. Total RNA was extracted using the Trizol reagent (Invitrogen, Life Technologies, USA) following the manufacturer’s instructions.

### cDNA library construction and RNA sequencing

A total of 1.5 µg of RNA per sample was used as the input material for the RNA sample preparations. Sequencing libraries were generated using NEBNext Ultra RNA Library Prep Kit for Illumina (NEB, USA) following the manufacturer’s recommendations, and index codes were added to attribute the sequences to each sample. The concentration and size of the cDNA library was determined with a Qubit 2.0 Fluorometer (Invitrogen, Carlsbad, CA, US), and library quality was assessed on the Agilent 2100 Bioanalyzer (Agilent Technologies, Santa Clara, CA, USA). After cDNA sample quality inspection, the library preparations were sequenced on an Illumina HiSeq. 2500 system (Illumina, San Diego, CA, USA) with paired-end reads. The base periods and mass fractions resulting from the high–throughput sequencing were recorded in the FASTQ format.

### *De novo* transcriptome assembly and functional annotation

The raw reads were filtered to remove adaptors, low-quality reads (less than 50% ratio of greater than 20 bases), and reads showing an N ratio (unknown sequences) greater than 5% using the FastX programme (version 0.0.13). After filtering, we obtained and evaluated the clean reads. A pool of reads was formed by merging eighteen samples of sequencing data. We performed *de novo* transcriptome assembly of the clean reads to obtain the final unigenes using Trinity^56^ with min_kmer_cov set to 2 by default and all other parameters set to default. Gene function was annotated based on the following databases: NR (NCBI non-redundant protein sequences), Nt (NCBI non-redundant nucleotide sequences), Swiss-Prot (A manually annotated and reviewed protein sequence database), PFAM (Protein family), KOG/COG (Clusters of Orthologous Groups of proteins), KEGG (Kyoto Encyclopedia of Genes and Genomes) and GO (Gene Ontology).

### Differentially expressed gene (DEG) profiling of rice and water-oat populations of *C. suppressalis*

By combining and splicing the mRNA sequences of the 18 samples from the two populations of *C. suppressalis*, we obtained transcriptome databases and used them as a reference sequence. We matched the clean reads of each sample in the reference sequence using RSEM software^57^. Default values were used for the Bowtie 2 parameters used in the RSEM software.

For the differential gene expression analysis, we obtained the key genes with differentially expressed counts from the different samples and performed GO functional analysis and KEGG pathway analysis. The *P-*value was calculated as described by Robinson and Rivals^58,59^. The FPKM (Fragments Per Kilobase of transcript sequence per Millions base pairs sequenced) method was used to calculate the unigene expression counts and differential expression levels between different samples for an individual gene^60^. This method eliminates the influence of differences in lengths and number of sequences on the expression level. In addition, we used DESeq to screen for differentially expressed genes among the two populations. Genes with a |log2 ratio| ≥ 1 and an adjusted *P*-value <0.05 were used as the parameters for DEGs.

### Identification of putative genes associated with sex pheromone biosynthesis and DEGs analysis

We focused on the putative target genes involved in moth sex pheromone production. Candidate unigenes were selected according to their NR annotation. To compare the expression levels of sex pheromone biosynthetic genes between rice and water-oat populations, a profiling analysis of the DEGs was conducted. The FPKM value of each gene was used for comparing expression levels.

### Quantitative real-time PCR (RT-qPCR) validation

To further validate the differences in gene expression levels between the rice and water-oat populations, we randomly selected 5 genes and quantified their expression by ABI QuantStudio 6 Flex (Thermo Fisher Scientific, Massachusetts, USA), with three biological and technical replicates. Briefly, total RNA was extracted using the RNAiso Plus reagent (TAKARA, 9109, Dalian, China). The PrimeScript RT Reagent Kit with gDNA Eraser (TAKARA, RR047, Dalian, China) were used to reverse transcribe and synthesize cDNA. Premier 5.0 (Premier Biosoft International, Palo Alto, CA) was used to design the primers (Table S4). The *β-actin* and *GAPDH* genes of *C. suppressalis* were used as internal reference genes^24^. The melting curves and standard curves of *β-actin*, *GAPDH*, *ELO1*, *ALF* and *ACD* were analyzed by qTOWER 2.0 Real-Time PCR (Analytik Jena, Jena, Germany), and *DES1* and *DES7* were determined by ABI QuantStudio 6 Flex (Fig. S2). RT-qPCR efficiency of primers were determined by a slope analysis with a linear regression model. Calculating the corresponding RT-qPCR efficiency (E) according to the following formula: E = (10^[−1/slope]^−1) × 100%^61^. Relative expression was analyzed via the 2^−ΔΔCT^ method^62^ and normalized the geometric mean of two reference genes expression^63,64^.

### Data analysis

Heatmap analysis is based on normalised FPKM via Z-score method^65^. The relative gene expression values were analysed by SPSS 11.5 software (SPSS Inc., Chicago, IL, USA, https://www.ibm.com/analytics/spss-statistics-software) with independent sample t-tests. All the results were visualised using GraphPad Prism 8 (GraphPad Software Inc., San Diego, CA, https://www.graphpad.com) software packages. The FPKM values and relative expression values were represented as the means ± standard error of mean (SEM) based on three biological replicates.

## Supplementary information

Supplementary material.

Supplementary material 2.

## Data Availability

All the data presented here will be available under request to the scientific community.
