# Supplementary material for: Comparative transcriptomics of the pheromone glands provides new insights into the differentiation of sex pheromone between two host populations of Chilo suppressalis
Source: Sci Rep. 2020 Feb 26;10:3499. doi: 10.1038/s41598-020-60529-x (PMC7044216; doi:10.1038/s41598-020-60529-x)
Supplement: Supplementary file 1 — Supplementary material. [file 41598_2020_60529_MOESM1_ESM.pdf]

Comparative transcriptomics of the pheromone glands provides new insights into the differentiation of sex pheromone between two host populations of *Chilo suppressalis*

Shuang Guo[#], Zhong Tian[#], Wei-Li Quan, Dan Sun, Wen Liu & Xiao-Ping Wang[*]
Hubei Key Laboratory of Insect Resources Utilization and Sustainable Pest Management, College of Plant Science & Technology, Huazhong Agricultural University, Wuhan, 430070, PR China
[#] These authors contributed equally to this work.
[*] Correspondence author.
Xiao-Ping Wang, xpwang@mail.hzau.edu.cn

Supplementary Figure

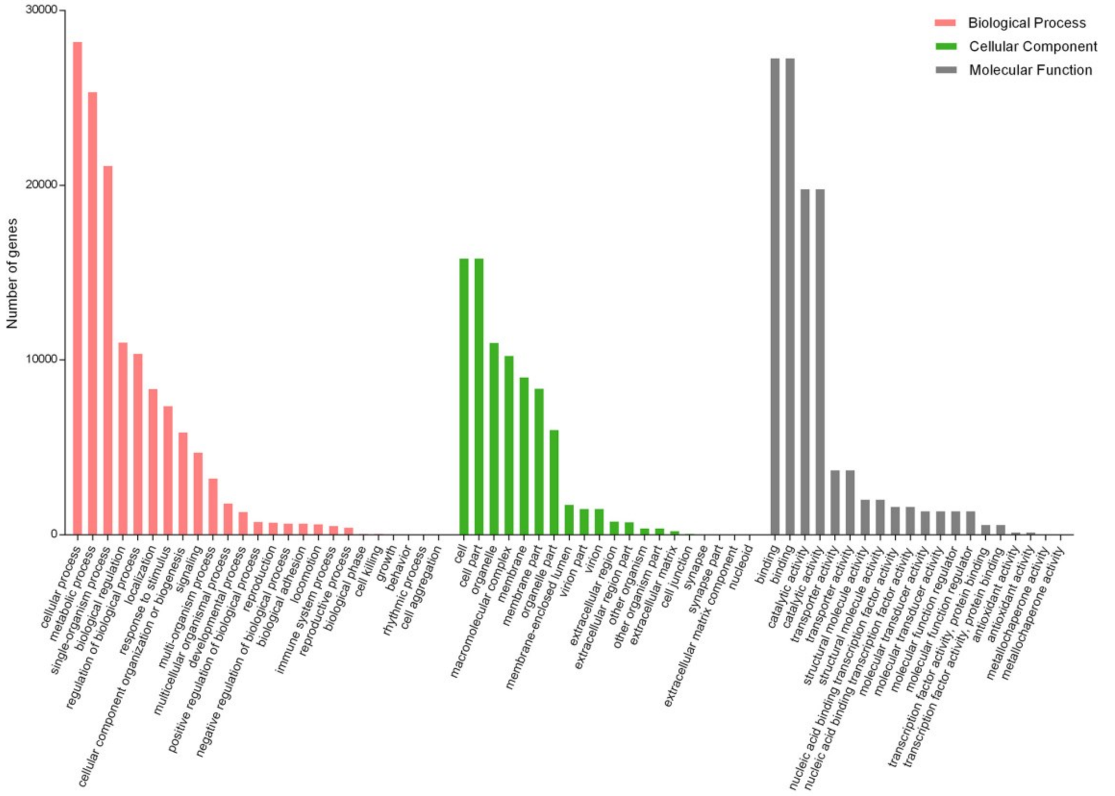

Figure S1. GO functional classifications of the transcriptomes of *C. suppressalis*. GO terms are determined using Blast2GO (version 2.5) software with an e-value cutoff of 10^{-6}. Then, the results were visualised by GraphPad Prism 8 (GraphPad Software Inc., San Diego, CA, https://www.graphpad.com).

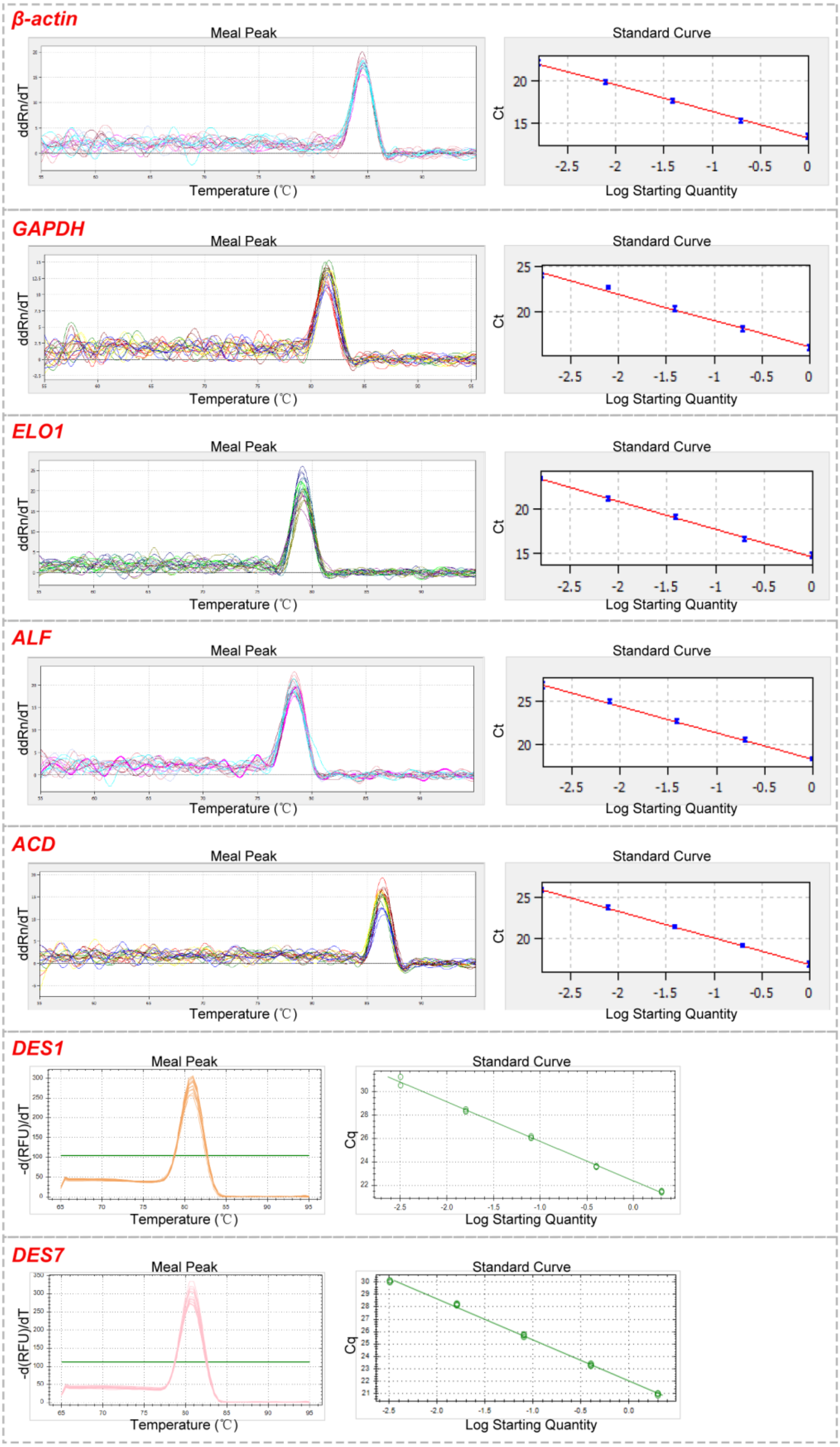

Figure S2. The melting curves and standard curves of RT-qPCR primers for verifying the specificity and determine the PCR efficiency

Supplementary Table

Table S1. Assembly of the sex pheromone gland transcriptome of *C. suppressalis*.

Length interval and statistics index	Transcripts	Unigenes
200-500bp	188,484	62,987
500-1kbp	54,264	51,372
1k-2kbp	30,520	30,491
>2kbp	24,627	24,627
Total number	297,895	169,477
Min length (bp)	201	201
Mean length (bp)	757	1,115
Median length (bp)	371	642
Max length (bp)	29,656	29,656
N50 length (bp)	1,383	1,830
N90 length (bp)	278	467
Total nucleotides	225,384,118	189,013,525

Table S2. Annotation results of the sex pheromone gland transcriptome of *C. suppressalis* in different databases.

Database	Number of Unigenes	Percentage (%)
Nr	61422	36.24
Nt	28805	16.99
KO	21036	12.41
SwissProt	42162	24.87
PFAM	47893	28.25
GO	48322	28.51
KOG	30528	18.01

Table S3. All DEGs between rice and water-oat populations.

Table S4. Primers for RT-qPCR.

Genes	Forward primers (5’-3’)	Reverse primers (5’-3’)	Amplicon size	PCR efficiency	Standard curve R^2
β-actin	gatcaagatcatcgcaccac	acttcctgtgcacgattgag	143 bp	109%	0.998
GAPDH	cactaccaactgccttgctc	gggtggcagtagtagcatga	98 bp	119%	0.992
ELO1	atgtcctcaccgtttccgac	gccgctgactatgctctcat	180 bp	111%	0.999
ALF	cgacatctaccgagaacgca	gttagaggcaggcgttgagt	110 bp	117%	0.999
ACD	gccgttagttgctgcatacg	ctctccacaatgaagccggt	222 bp	103%	0.999
DES1	acaactatcaccacacctttcca	ccgtcaccagtacgttttactcta	172 bp	98%	0.997
DES7	tgaaacaggccgctcagaaa	acgtgaacgcaaggtacagt	105 bp	101%	0.998
